# Tensile Properties and Thermal Stability of Unidirectionally <111>-Oriented Nanotwinned and <110>-Oriented Microtwinned Copper

**DOI:** 10.3390/ma13051211

**Published:** 2020-03-08

**Authors:** Yu-Jin Li, King-Ning Tu, Chih Chen

**Affiliations:** 1 Department of Materials Science and Engineering, National Chiao Tung University, Hsinchu 30010, Taiwan; r777719982003@yahoo.com.tw; 2 Department of Materials Science and Engineering, University of California at Los Angeles, Los Angeles, CA 90095, USA; kntu@ucla.edu; 3 International College of Semiconductor Technology, National Chiao Tung University, Hsinchu 30010, Taiwan

**Keywords:** nano-twinned Cu, toughness, thermal stability, electroplated Cu

## Abstract

Tensile tests on two kinds of electroplated copper foils with twins before and after annealing were performed. One electroplating parameter results in a microstructure of <110>-oriented microtwinned Cu (mt-Cu), and the other is <111>-oriented nanotwinned Cu (nt-Cu). The latter shows higher thermal stability than the former after annealing. Though the toughness for the two as-plated foils are quite close, the toughness for the <111> oriented nt-Cu increased from 34 to 74 MJ/m^3^ after annealing at 250 °C for 3 h. In comparison, the toughness of the <110>-oriented mt-Cu remained almost the same after annealing.

## 1. Introduction

Copper is a vital interconnect material in the semiconductor industry because of its low resistance, and excellent filling capability in through silicon via and trenches. As the requirements of microelectronic devices continue to increase, the dimension of Cu conducting lines will continue to shrink. With higher interconnect density in next-generation electronic devices, the width of Cu lines have decreased to 2 μm, and will continue to reduce to submicron parameters in 2022 [1]. In addition, the chip requirement and chip count for artificial intelligence (AI) continues to increase, with the package size increasing to 70 × 70 mm. However, the Cu lines may fail due to severe mechanical stress in large packages. An example is the use of the Cu redistribution layer (RDL) in the integrated fan-out (InFO) wafer-level packages [2,3]. Cu lines are electroplated for the interconnects. The Cu RDL needs to be passivated by polyimide (PI), and the PI curing is performed at 230 °C for 1 h. If there are three layers of RDL, the first Cu RDL will experience three curing processes of PI (i.e., 250 °C for a total of 3 h). Therefore, the Cu RDL needs to be thermally stable up to 250 °C for 3 h and possess high toughness to avoid fracture.

In recent years, nanotwinned copper (nt-Cu) was found to have high strength, ideal ductility, excellent thermal stability, low resistivity [4,5], high electromigration resistance [6], and history-independent cyclic response during fatigue tests [7,8]. Generally, magnetron sputtering and electroplating have been applied in fabricating highly <111>-oriented nt-Cu. In 2008, Anderoglu et al. [9] reported that columnar nt-Cu film with a strong <111> texture can be fabricated by magnetron sputtering, with an average grain size of up to 50 nm and nanotwin twin spacing of 16 nm. The columnar nt-Cu grain was thermally stable after annealing at 800 °C for 1 h. The results indicated that the sputtered highly <111>-textured columnar grain with high-density nanoscale coherent twins is thermally stable. In 2008, Hodge et al. reported a yield peak in the stress–strain curve of nanotwinned Cu film; with 170 nm-thick nt-Cu foils and twin density higher than 3 × 10^6^ (m^2^/m^3^) [10,11]. In 2012, Liu et al. succeeded in depositing <111>-textured nt-Cu by direct current electroplating. There are many incoherent nanotwins in electroplated Cu, and it is reported that incoherent nanotwins are not thermally stable [12].

Electroplated Cu is known to have very low-thermal stability. Electroplated Cu films undergo grain growth even at room temperature, referred as self-annealing [13]. Nano-twinned Cu possesses high strength and ideal ductility, thus it has potential to be adopted in RDLs in the packaging industry. The mechanical properties of nt-Cu has been reported in many previous studies [4,5,10,11,14,15]. Nevertheless, reports on the temperature-dependent tensile property of nt-Cu are still lacking.

In this study, we examine DC-electroplated nt-Cu films with different twin structures before and after annealing at 250 °C, to evaluate its thermal stability and mechanical properties. Tensile tests are performed to observe the mechanical properties of different microstructures. We aim to apply our findings in the InFO RDLs to solve the softening issues during manufacturing processes and tensile stress during operation.

## 2. Materials and Methods

We electroplated columnar nt-Cu films on Si substrates with layers of 5 μm PI, 100 nm Ti, and a 200 nm Cu seed layer. The plating bath consisted of high-purity copper sulfate, with the concentration of Cu^2+^, H_2_SO_4_, and Cl^−^ controlled to be 50 g/L, 50 g/L, and 50 ppm, respectively. To fabricate <111> nt-Cu, a disclosed additive was added. Another set of reference specimens was also electroplated at 40 mA/cm^2^ without the additive. They are denoted as <110>-oriented micro-twinned Cu (mt-Cu). The copper films were electroplated by direct current with a current density of 40 mA/cm^2^ for 30 min. Electro-polishing was applied to reduce the morphological defects and the surface roughness. The electro-polishing bath consisted of phosphoric acid, acetic acid, performed with an inert anode. The thickness of Cu film after the polishing process was approximately 20 μm. After the electropolishing process, the Cu films were peeled off from the Si substrates. Due to the poor adhesion between the PI layer and the Si substrate, the free-standing Cu foils contained a thin Ti and Cu seed layer. Compared to the thickness of the Cu foils, the Ti and Cu seed will not affect the measured mechanical properties of the Cu foils.

**Figure 1:**
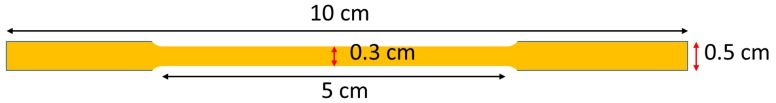
The schematic figure of the tensile specimen.

To examine the thermal stability of the copper foils, the polished copper foils were annealed at 250 °C for 10 min and 3 h before the tensile test. The gauge length of the specimen was 5 cm and the width was 3 mm, as shown in Figure 1. After the sample preparation, the microstructure and thickness were observed with a dual beam system (focused ion beam and electron beam, FEI Nova 200, Brno–Kohoutovice, Czech Republic). The ion beam energy was 30 kV with a current of 11 nA to etch the surface to expose the microstructure. The tensile tests were performed with an electro mechanical tension/compression frame (Instron 4465, Norwood, MA, USA). The experiments were conducted at room temperature with a strain rate of 4.3 × 10^−3^ (1/s). In order to ensure the accuracy of the test results, we tested at least 3 samples under each heat treatment condition.

The top view grain orientation maps were examined by electron backscattered diffraction (EBSD) with an Oxford system equipped in a Scanning electron microscope (SEM, JEOL JSM-7800F, Akishima, Tokyo, Japan), and was examined with an accelerating voltage of 20 kV, specimen tilt angle of 70°, step size from 0.02 to 1 µm for different levels of magnification. TSL OIM analysis was employed for further analysis of the crystallography of the specimen and measurement of average grain size. The tolerance angle was 15° and minimum grain size was set to 50 nm.

Field Emission Transmission Electron Microscope (FE-TEM, JEM-F200, Akishima, Tokyo, Japan) was employed to measure the average twin spacing.

## 3. Results and Discussion

**Figure 2:**
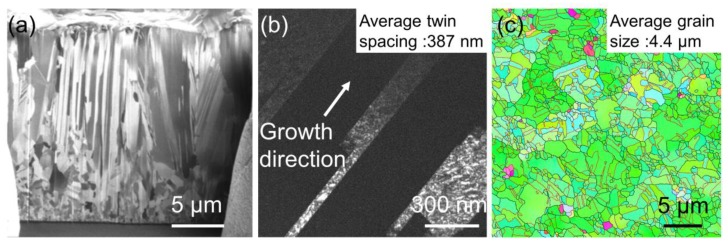
Microstructure of the copper foils. These figures show (**a**) the cross-sectional ion images; (**b**) TEM image of of <110>-oriented mt-Cu foil, average twin spacing of 387 nm; (**c**) plane view electron backscattered diffraction (EBSD) of <110>-oriented mt-Cu foil, average grain size of 4.4 μm. Low-angle grain boundary, high-angle grain boundary, and twin boundary are marked in blue lines, black lines, and red lines, respectively.

**Figure 3:**
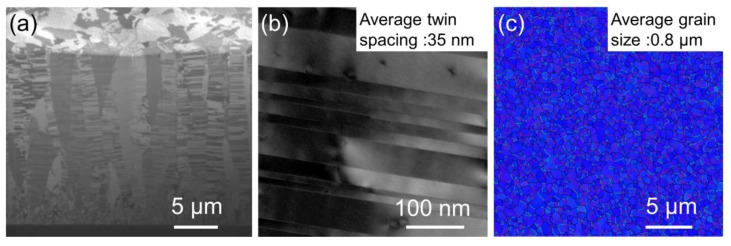
Microstructure of the copper foils. These figures show (**a**) the cross-sectional ion images; (**b**) TEM image, average twin spacing of 35 nm; (**c**) plane view EBSD of <111>-oriented nt-Cu foil, average grain size of 0.8 μm. Low-angle grain boundary, high-angle grain boundary, and twin boundary are marked in green lines, black lines, and red lines, respectively.

Two kinds of columnar twinned structures were fabricated: <110>-oriented mt-Cu and <111>-oriented nt-Cu. Figure 2a shows the cross-sectional FIB image for as-fabricated <110>-oriented mt-Cu film on Si substrate. Twins are observed in the 5 μm thick films, with the average twin spacing measured to be 387 nm, as shown in Figure 2b. The direction of twin planes is highly parallel to the growth direction of the Cu film. In addition, the Cu film possesses a high <110> preferred orientation, as shown in the plan-view EBSD orientation in Figure 2c. Approximately 91% of the top surface area was <110>-oriented grains, and the average grain size was measured to be 4.4 μm. Low-angle grain boundary, high-angle grain boundary and twin boundary are shown in Figure 2c with blue, black and red lines. The other specimen was <111>-oriented nt-Cu, as illustrated in Figure 3a, in which densely-packed nanotwinned microstructure was observed. The average twin spacing was measured to be 35 nm (as shown in Figure 3b), and the twin plane direction was vertical to the growth direction. Columnar grains grew after the formation of approximately 2 μm-thick nanocrystalline grains, which is referred to as the transition layer. Figure 3c presents the orientation analysis of the surface grains, with 100% of the surface being <111>-oriented with an average grain size of 0.8 μm. Low-angle grain boundary, high-angle grain boundary and twin boundary of <111>-oriented nt-Cu are shown in Figure 3c with green, black, and red lines, respectively. The number of twin boundaries on the surface of <111>-oriented nt-Cu is much less than those on <110>-oriented mt-Cu, because most of the twin boundaries in <111>-oriented nt-Cu were parallel to the surface.

**Figure 4:**
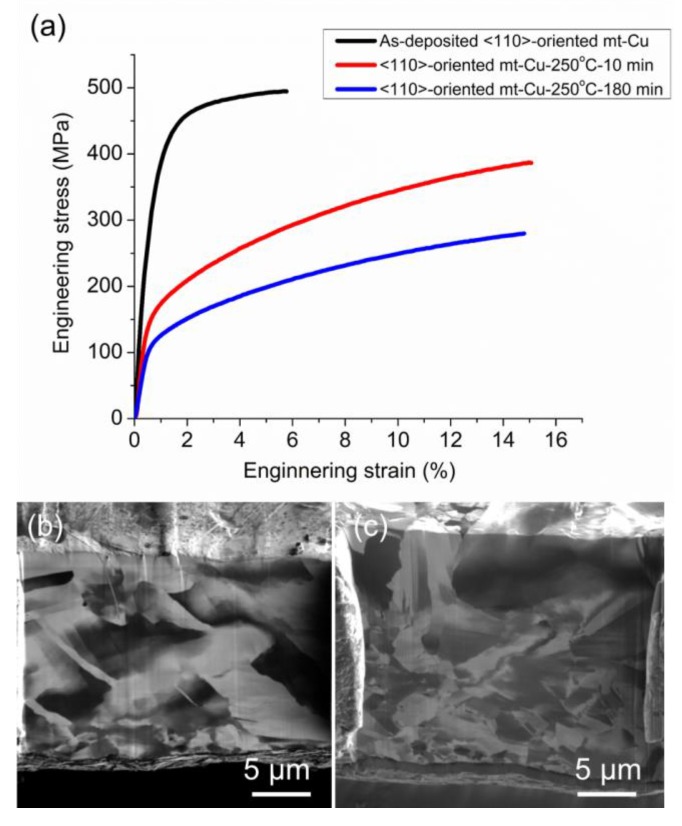
(**a**) The stress–strain curve of <110>-oriented mt-Cu foils before and after annealing at 250 °C for 10 and 180 min. The cross-sectional ion images of mt-Cu after annealing at 250 °C for (**b**) 10 min and (**c**) 180 min.

The <110>-oriented as-fabricated mt-Cu foil had high tensile strength, but it decreases dramatically after annealing. Figure 4a depicts the engineering stress–strain curves for the as-fabricated, 250 °C—10 min, 250 °C—180 min annealed <110> mt-Cu foils. The 0.2% offset yield strength for the as-fabricated foil was 412 MPa, and the elongation was only 6%. However, the yield stress dropped abruptly to 131 MPa after annealing at 250 °C for 10 min, and it further decreased to 107 MPa after annealing at 250 °C for 180 min. The elongation increased to about 15% after annealing at 250 °C. Microstructure analysis was performed to determine the reason for the drop in yield strength after annealing. Figure 4b,c shows the cross-section FIB image for the Cu foils after the 250 °C—10 min and 250 °C—180 min annealing. Significant grain growth took place after annealing at 250 °C for 10 min, and the micro-scale twins disappeared. The grain size increased after annealing at 250 °C for 180 min, as seen in Figure 4c. It is reported that the twin boundaries can effectively strengthen materials [4,5]. Grain boundaries are able to impede the movement of dislocations, and thus strengthen the metals. In the <110> mt-Cu foils, thermal annealing annihilated both twin and grain boundaries simultaneously, and weakened the <110>-oriented mt-Cu structure.

**Figure 5:**
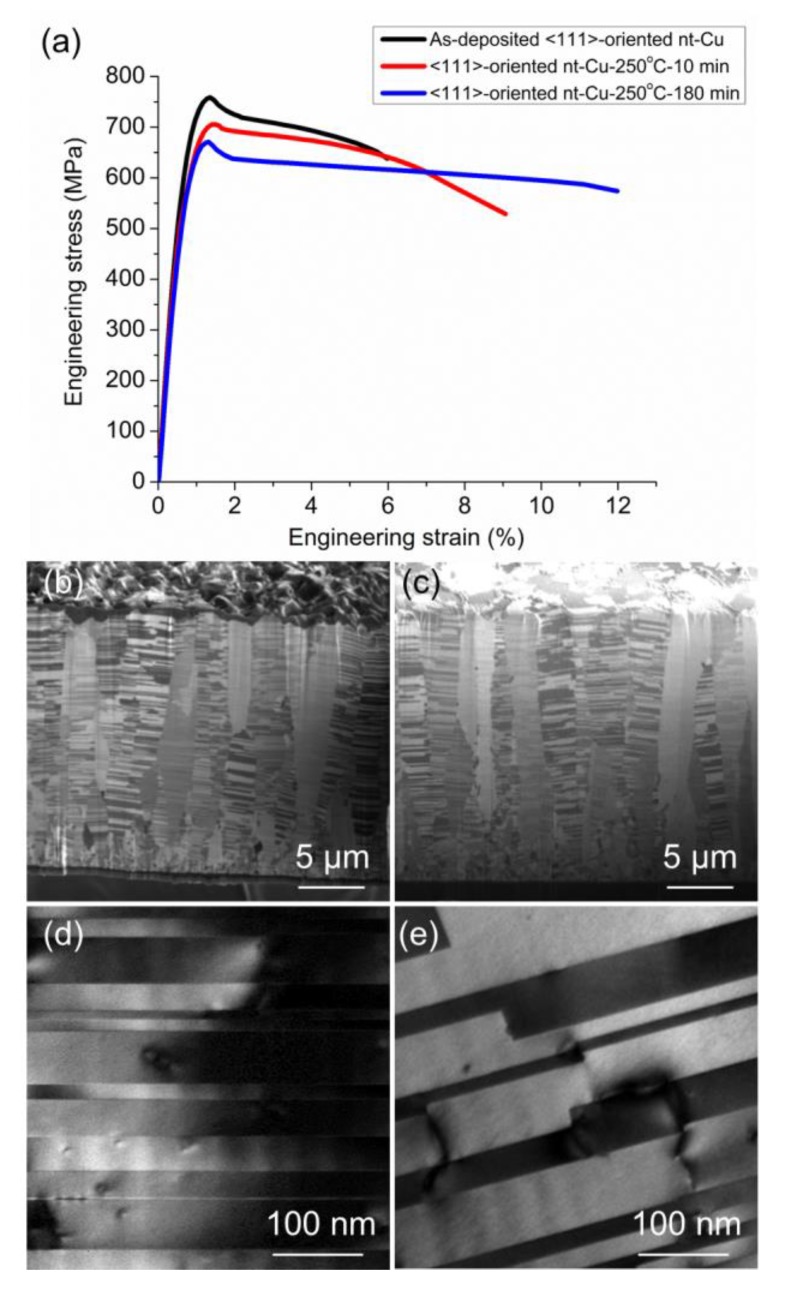
(**a**) The stress–strain curve of <111>-oriented nt-Cu foils before and after annealing at 250 °C for 10 and 180 min. The cross-sectional ion images of nt-Cu after annealing at 250 °C for (**b**) 10 min and (**c**) 180 min. (**d**,**e**) are the TEM images of <111> nt-Cu.

However, the <111>-oriented nt-Cu film exhibits high-thermal stability and high-mechanical strength after annealing at 250 °C. Figure 5a presents the measured engineering stress–strain relation for the as-electroplated, 250 °C—10 min and 250 °C—180 min annealed <111> nt-Cu foils. The thickness of the Cu foils was almost the same as that of <110> mt-Cu foils. The yield strength was high as 748 MPa for the as-deposited nt-Cu foil, and the elongation was only 6%. After the annealing at 250 °C for 10 min, the yield strength dropped slightly to 700 MPa, but the elongation increased to 9%. Upon annealing at 250 °C for 180 min, the strength decreased slightly to 670 MPa, yet the elongation further increased to 12%. The elongation was almost comparable to the <110> mt-Cu, but the yield strength was approximately six times higher than that of the mt-Cu after the annealing at 250 °C for 180 min. The main reason for the high strength after annealing is attributed to the high-thermal stability of the columnar <111>-oriented nt-Cu grains. Figure 5b shows the cross-sectional microstructures for the nt-Cu foil after annealing at 250 °C for 10 min. No obvious grain growth was observed, except that some of the nanocrystalline grains at the bottom of the film were consumed by the <111>-oriented nt-Cu columnar grains. Figure 5c shows the microstructures of nt-Cu grains after annealing at 250 °C for 180 min. Columnar grains with densely-packed nanotwins were still intact after the heat treatment. The average twin spacing of <111>-oriented nt-Cu increased to 43 and 46 nm after annealing at 250 °C for 10 min and 3 h (Figure 5d,e). The microstructure did not change significantly, even after the long annealing session at 250 °C. Therefore, the yield strength only dropped slightly. For all <111>-oriented nt-Cu samples, the engineering stress decreased during the tensile test due to the difference of required stress for dislocation nucleation and motion with low-dislocation density [10].

**Figure 6:**
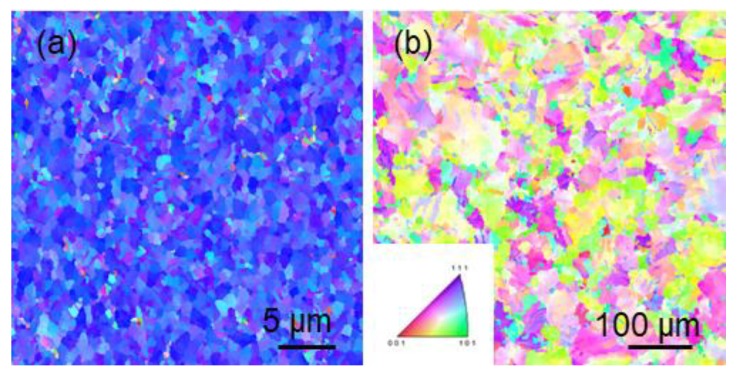
The grain orientation image maps of (**a**) <111>-oriented nt-Cu, and (**b**) <110>-oriented mt-Cu after annealing at 250 °C for 10 min.

**Figure 7:**
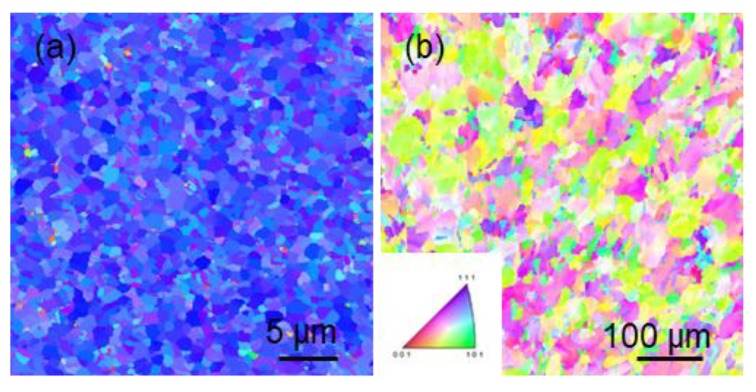
The grain orientation image maps of (**a**) <111>-oriented nt-Cu, and (**b**) <110>-oriented mt-Cu after annealing at 250 °C for 180 min.

In addition, we can observe from Figure 6 and Figure 7 that the thermal stability of <110> preferred oriented nt-Cu is inferior to that of <111> preferred oriented nt-Cu. Researches have been performed on the thermal stability of <110>-oriented coherent twin boundaries [16], where the presence of silicon substrate induced thermal stress due to the thermal expansion coefficient difference between Cu/Si during annealing. The thermal stress drives incoherent twin boundaries to slip, causing a detwinning phenomenon in coherent twin boundaries. In our experiment, the copper foil was a freestanding structure, and the annealing process was in a nitrogen atmosphere where heat conduction was uniform. The annealing process would not generate thermal stress. We speculate that since electroplated copper has high-thermal stability on its own, along with the internal stress formed during electroplating, recrystallization and grain growth is likely to occur during annealing. From Figure 6a, we can observe that the post-annealed <111> copper foil does not show obvious orientation change at 250 °C for 10 min, yet in Figure 6b the <110> copper foil has a completely different surface orientation after the annealing process. The original 90% <110> preferred orientation is fully replaced by randomly-oriented micron-scale large grains. In Figure 7a we can see that the post-annealed <111> copper foil still shows no orientation change at 250 °C for 180 min. From Figure 7b we can observe that annealing for 180 min does not result in a big difference from annealing for 10 min in the <110>-oriented mt-Cu. This issue has not been fully examined in past researches. Detwinning caused by dislocation slip due to the absence of thermal stress is an unsuitable explanation for this scenario, yet detwinning is an urgent issue to be addressed. On the other hand, the results highlight the advantages of high-thermal stability in <111>-oriented nt-Cu films.

**Figure 8:**
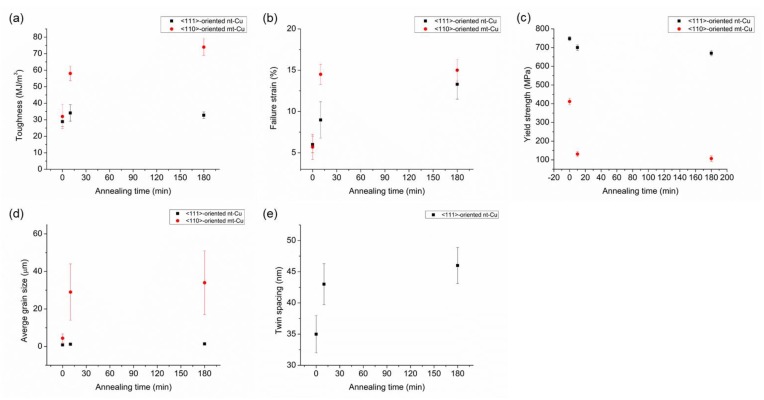
The (**a**) toughness, (**b**) failure strain, (**c**) yield strength, and (**d**) average grain size of <110>-oriented mt-Cu and <111>-oriented nt-Cu before and after annealing at 250 °C for 10 and 180 min. (**e**) The average twin spacing of <111>-oriented nt-Cu.

Toughness serves as an important index for the redistribution lines in fan-out packaging, as previous researches have indicated that copper lines will be under constant tension and cause crack formation [17]. It represents the energy absorbed upon fracture per unit volume in materials, which is also the area under the stress–strain curve. Figure 8a shows the measured toughness for the specimens. For the <110> mt-Cu sample, the toughness is 28.9 MJ/m^3^ for the as-fabricated foil, and increases slightly to 34.1 MJ/m^3^ after annealing at 250 °C for 10 min. It decreases to 32.7 MJ/m^3^ after annealing at 250 °C for 180 min. However, the toughness of the <111> nt-Cu increases significantly as the annealing time increases at 250 °C. The toughness for the as-deposited nt-Cu is 32 MJ/m^3^. It rises abruptly to 58 MJ/m^3^ after annealing at 250 °C for 10 min, and it further increases to 74 MJ/m^3^ as the annealing time prolongs to 180 min. The yield strength, failure strain, average grain size, and twin spacing of the Cu foils after different annealing time are shown in Figure 8b–e. The yield strength drops significantly in <110> mt-Cu after annealing at 250 °C for 10 min. It could be attributed to serious de-twinning and large grain growth (Figure 8d). In contrast, the twin spacing in <111>-oriented nt-Cu is relatively stable. Moreover, the increase in elongation reflects that the elimination of fine grain at the bottom of the nt-Cu foils during annealing is very helpful to the elongation.

**Figure 9:**
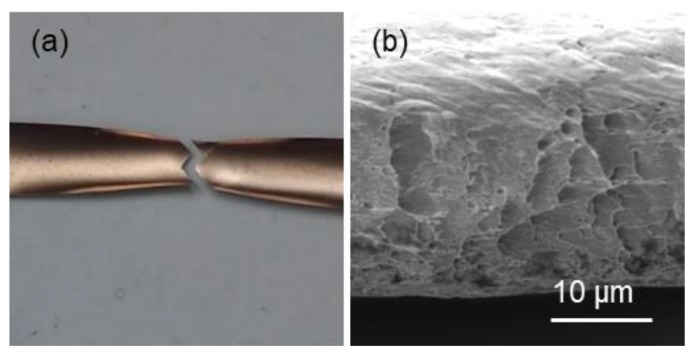
The (**a**) necking behavior and (**b**) fracture images of <111>-oriented nt-Cu after annealing at 250 °C for 180 min.

The significant increase in toughness after annealing is attributed to two reasons. First, the <111>-oriented columnar nt-Cu grains are quite stable, and the strength of the nt-Cu maintains at about 650 MPa after annealing. Huang et al. reported that the columnar grain grew downward to consume the tiny grains at the bottom of the Cu film after annealing at 250–300 °C, and nanotwins grew in the original tiny grain regions [18]. This is because the nanotwins intersect the grain boundaries of columnar grains and modified the grain boundary structures to stabilize the columnar grains. However, for <110>-oriented mt-Cu, the twin planes are almost parallel to the Cu grain boundaries. The Cu grain boundaries were not stabilized by the microtwins. Thus upon annealing at 250 °C, most of the Cu grain boundaries and microtwins in were consumed by grain growth. Second, the <111>-oriented nt-Cu become ductile after the annealing at 250 °C, although there was no significant grain growth in the columnar grains. Figure 9 shows the necking behavior and SEM image for the fractured foil annealed at 250 °C for 180 min. Obvious necking and ductile fracture were observed. However, grain growth happened in the nanocrystalline layer at the bottom of the nt-Cu film. It is reported ultra-fine grain can strengthen the Cu film, but also result in the brittleness of the Cu film. As shown in Figure 5a,b, the nanocrystalline layer became thinner than the as-fabricated specimen, and nanotwin formation also took place in the layer. Therefore, the elongation of the nt-Cu foils increased significantly after the annealing at 250 °C.

It is noteworthy that the tensile properties are different from fatigue properties. Yet, they are related to some extent [19]. Metallic materials with a high tensile strength might have a low-fatigue lifetime. However, the nanotwinned Cu films have been demonstrated to have high-fatigue resistance [8]. In addition, Li et al. also reported nanotwinned Cu redistribution lines possess high-fatigue resistance to temperature cycling tests [20]. Therefore, the nanotwinned Cu may have the potential to be adopted for redistribution lines for next-generation packaging.

## 4. Conclusions

The toughness of the electroplated and unidirectionally-oriented nt-Cu increased by two-fold after post-annealing at 250 °C for 180 min, because of the high-thermal stability of the unidirectional twinning structure and the consumption of the nanocrystalline grains. In contrast, the <110>-oriented mt-Cu is thermally unstable; therefore, the tensile stress dropped drastically from nearly 500 to 300 MPa after annealing at 250 °C. We note that, with the shrinking of fine-pitch interconnects, the toughness and the thermal stability of Cu RDL in microelectronic devices are of critical concern. In this study, we show that the electroplated nt-Cu provides an excellent solution to solve both issues.
